# Advance Care Planning in the Context of COVID-19: Complexities Across a Range of Constituents

**DOI:** 10.1093/geroni/igab046.511

**Published:** 2021-12-17

**Authors:** Brian Carpenter, Karen Hirschman

## Abstract

The COVID-19 pandemic brought serious illness and death into close proximity for a large number of people, whether through personal experience, infection in family members or friends, or unremitting media coverage on the effects of the virus and widespread mortality. Because of a collective vulnerability to illness and the heightened possibility of death, more people began to contemplate what kinds of medical care they would want if they ever became seriously ill. In other words, more people began the process of advance care planning (ACP). This symposium explores how the COVID-19 pandemic shifted interest in and execution of ACP across a range of groups. The first presentation reviews survey data from a large, community-based sample of older adults about their ACP conversations before and after the start of the pandemic. Shifting to the experience of clinicians, the second presentation summarizes a survey with multidisciplinary healthcare professionals about ACP conversations in their personal lives during the pandemic and how their observations of patients influenced their own plans. The third presentation describes the reactions of undergraduate students to an ACP class exercise, including COVID-19 as a motivating factor for pursuing ACP. The final presentation concludes with a review of two clinical cases that illustrate how COVID-19 has upended traditional ACP and highlighted the need for new policies and processes, with a particular focus on ethics and equity. Together, these presentations offer diverse insights into how ACP may shift in a post-pandemic world.

